# *IRAK1* Duplication in *MECP2* Duplication Syndrome Does Not Increase Canonical NF-κB–Induced Inflammation

**DOI:** 10.1007/s10875-022-01390-7

**Published:** 2022-11-02

**Authors:** Ilona Gottschalk, Uwe Kölsch, Dimitrios L. Wagner, Jonas Kath, Stefania Martini, Renate Krüger, Anne Puel, Jean-Laurent Casanova, Aleksandra Jezela-Stanek, Rainer Rossi, Salima El Chehadeh, Hilde Van Esch, Horst von Bernuth

**Affiliations:** 1 Department of Pediatric Respiratory Medicine, Immunology and Critical Care Medicine, Charité-Universitätsmedizin Berlin, corporate member of Freie Universität Berlin, Humboldt-Universität zu Berlin, and Berlin Institute of Health (BIH), Berlin, Germany; 2 Berlin-Brandenburg Center for Regenerative Therapies (BCRT), Berlin Institute of Health (BIH), Charité-Universitätsmedizin Berlin, Augustenburger Platz 1, 13353 Berlin, Germany; 3 Labor Berlin GmbH, Department of Immunology, Berlin, Germany; 4 Berlin Center for Advanced Therapies (BeCAT), Charité-Universitätsmedizin Berlin, Corporate Member of Freie Universität, Berlin, Humboldt-Universität Zu Berlin, and Berlin Institute of Health (BIH), Berlin, Germany; 5 Institute of Transfusion Medicine, Charité-Universitätsmedizin Berlin, corporate member of Freie Universität Berlin, Humboldt-Universität zu Berlin, and Berlin Institute of Health (BIH), Berlin, Germany; 6 Institute of Medical Immunology, Charité-Universitätsmedizin Berlin, corporate member of Freie Universität Berlin, Humboldt-Universität zu Berlin, and Berlin Institute of Health (BIH), Campus Virchow-Klinikum, Berlin, Germany; 7 Laboratory of Human Genetics of Infectious Diseases, Necker Branch, INSERM U1163, Necker Hospital for Sick Children, Paris, France; 8 Imagine Institute, University of Paris, Paris, France; 9 St. Giles Laboratory of Human Genetics of Infectious Diseases, Rockefeller Branch, The Rockefeller University, New York, NY USA; 10 Howard Hughes Medical Institute, New York, NY USA; 11 Pediatric Hematology and Immunology Unit, Necker Hospital for Sick Children, AP-HP Paris, France; 12 Department of Genetics and Clinical Immunology, National Institute of Tuberculosis and Lung Diseases, Warsaw, Poland; 13 Childrens’ Hospital Neukölln, Vivantes GmbH, Berlin, Germany; 14 Institute of Medical Genetics of Alsace (IGMA), Strasbourg, France; 15 Center for Human Genetics, University Hospitals Leuven, Louvain, Belgium; 16 Berlin Institute of Health, Charité-Universitätsmedizin Berlin, Berlin, Germany

**Keywords:** Xq28 Duplication syndrome, Methyl CpG binding protein 2 (*MECP2*) duplication syndrome, Methyl CpG binding protein 2 (MECP2), Interleukin-1 receptor–associated kinase 1 (IRAK1), Canonical NF-κB signaling, Inborn errors of immunity

## Abstract

**Purpose:**

Besides their developmental and neurological phenotype, most patients with *MECP2/IRAK1* duplication syndrome present with recurrent and severe infections, accompanied by strong inflammation. Respiratory infections are the most common cause of death. Standardized pneumological diagnostics, targeted anti-infectious treatment, and knowledge of the underlying pathomechanism that triggers strong inflammation are unmet clinical needs. We investigated the influence of IRAK1 overexpression on the canonical NF-κB signaling as a possible cause for excessive inflammation in these patients.

**Methods:**

NF-κB signaling was examined by measuring the production of proinflammatory cytokines and evaluating the IRAK1 phosphorylation and degradation as well as the IκBα degradation upon stimulation with IL-1β and TLR agonists in SV40-immortalized fibroblasts, PBMCs, and whole blood of 9 patients with *MECP2/IRAK1* duplication syndrome, respectively.

**Results:**

Both, *MECP2/IRAK1*-duplicated patients and healthy controls, showed similar production of IL-6 and IL-8 upon activation with IL-1β and TLR2/6 agonists in immortalized fibroblasts. In PBMCs and whole blood, both patients and controls had a similar response of cytokine production after stimulation with IL-1β and TLR4/2/6 agonists. Patients and controls had equivalent patterns of IRAK1 phosphorylation and degradation as well as IκBα degradation upon stimulation with IL-1β.

**Conclusion:**

Patients with *MECP2/IRAK1* duplication syndrome do not show increased canonical NF-κB signaling in immortalized fibroblasts, PBMCs, and whole blood. Therefore, we assume that these patients do not benefit from a therapeutic suppression of this pathway.

**Supplementary Information:**

The online version contains supplementary material available at 10.1007/s10875-022-01390-7.

## Introduction

Patients with duplication of *methyl CpG binding protein 2 (MECP2)* on chromosome Xq28 were first described in 2005 [1, 2]. The clinical phenotype is characterized by developmental delay, hypotonia, epileptic seizures, as well as recurrent infections [1, 2]. Approximately 1% of severe X-linked intellectual disability in males might be explained by *MECP2* duplication syndrome (MDS) [3]. Reviewing the literature, we identified 102 articles describing patients with duplications in Xq28 of varying sizes but encompassing at least the *MECP2* and *interleukin-1 receptor–associated kinase 1* (*IRAK1)* gene, 14 of them published before the initial description of MDS (Table 1). From 1987 until now, at least 545 cases with confirmed genotype were published (504 males and 41 symptomatic females) (Table 1). Additionally, the duplication was suspected in 39 related patients (Table 1). However, the numbers of patients might be underestimated regarding the unevenly distributed origin of publications (43 European, 25 North American, 23 East-Asian, 5 rest of Asia including Russia, 3 Australian, 2 South American, 1 African) (Table 1). Most females with *MECP2* duplication are unaffected carriers showing a favorably skewed X chromosome inactivation (XCI) pattern [1, 4–6]. However, some females show a mild to severe phenotype. The main hypotheses are that symptoms in females might be caused, on the one hand, by the location of the duplicated material into an autosome or, on the other hand, by an unfavorable skewed X chromosome inactivation (XCI) [7–20]. However, the extent of the symptoms in females with *MECP2* duplication cannot be correlated with their XCI pattern, at least as assessed in peripheral blood [21].

**Table 1:** Overview of published patients suffering from *MECP2* duplication syndrome. We included all publications describing patients with duplication or triplication of at least *MECP2* and *IRAK1*. Also, we included publications describing patients with duplications in the region which included *IRAK1* although this gene was not described by then. Republished cases were only counted once as far as traceable. Note: Throughout the publications, the criteria for intellectual disability and developmental delay differ a lot. ND, not determined

Reference	Country	Published patients	Thereof male	Thereof female	Relatives with suspected disease	Intellectual disability or developmental delay	Infantile hypotonia	Epileptic seizures	Recurrent or severe infections	Thereof respiratory infections
Mohandas 1987 [22]	USA	1	1			1/1	ND	ND	ND	ND
Bertini 1992 [23]	Italy	2	2		5	2/2	2/2	2/2	2/2	2/2
Lahn 1994 [24]	USA	3	3			3/3	3/3	3/3	ND	ND
Vasquez 1995 [25]	Mexico	2	2		1	2/2	2/2	ND	2/2	2/2
Pai 1997 [26]	USA	5	5		1	5/5	ND	4/5	4/4	4/4
Goodman 1998 [27]	USA	4	4		1	4/4	4/4	1/4	2/4	2/2
Lubs 1999 [7]	USA	5	5			5/5	5/5	2/5	5/5	5/5
Lammer 2001 [28]	USA	1	1		3	1/1	1/1	ND	1/1	1/1
Akiyama 2001 [29]	Japan	1	1			1/1	1/1	1/1	1/1	1/1
Bialer 2003 [7]	USA	1		1		1/1	1/1	ND	1/1	1/1
Novelli 2004 [30]	Italy	1	1			1/1	1/1	ND	1/1	1/1
Kokalj-Vokac 2004 [31]	Slovenia	1	1			1/1	1/1	0/1	1/1	1/1
Lachlan 2004 [8]	UK	2	1	1		2/2	2/2	0/2	2/2	1/1
Teek 2004 [32]	Estonia	1	1			1/1	ND	ND	1/1	1/1
Meins 2005 [2]	Germany	1	1			1/1	1/1	1/1	1/1	1/1
Sanlaville 2005^a^ [9]	France	2	1	1		2/2	2/2	0/2	2/2	2/2
Van Esch 2005 [1]	Belgium	13	13			12/12	11/11	4/9	5/9	5/5
Friez 2006^a^ [33]	USA	13	13			13/13	12/13	9/13	13/13	12/12
Rosenberg 2006 [34]	Brazil	1	1		2	1/1	ND	ND	ND	ND
del Gaudio 2006 [4]	USA	7	7			7/7	7/7	1/7	4/7	4/4
Froyen 2007^b^ [35]	Belgium									
Madrigal 2007 [36]	Spain	1	1		2	1/1	1/1	ND	1/1	1/1
Bauters 2008^a^ [37]	France	4	4			ND	ND	ND	1/1	1/1
Smyk 2008 [38]	Poland	3	3		2	3/3	3/3	1/3	3/3	2/2
Lugtenberg 2009 [6]	Netherlands	13	13		1	13/13	13/13	7/13	3/13	3/3
Clayton-Smith 2009 [5]	UK	15	15			14/15	12/15	8/15	14/15	14/14
Prescott 2009 [39]	Norway	2	2			2/2	ND	1/2	2/2	2/2
Ramocki 2009^a^ [40]	USA	8	8		5	8/8	8/8	4/8	6/8	6/6
Velinov 2009 [41]	USA	1	1			1/1	1/1	0/1	0/1	ND
Echenne 2009 [42]	France	5	5			5/5	5/5	4/5	0/5	ND
Kirk 2009 [43]	Australia	3	3			3/3	1/1	2/2	2/2	2/2
Bartsch 2010 [44]	Germany	4	4			4/4	4/4	2/4	4/4	2/2
Honda 2010^c^ [45]	Japan									
Belligni 2010 [46]	Italy	1	1			1/1	1/1	ND	1/1	1/1
Reardon 2010 [12]	Ireland	8	7	1		8/8	2/2	4/8	7/8	7/7
Auber 2010 [10]	Germany	1		1		1/1	1/1	1/1	1/1	1/1
Campos 2010 [47]	Brazil	3	3			3/3	1/1	2/2	ND	ND
Fernandez 2010 [48]	Spain	1	1		1	1/1	1/1	1/1	1/1	1/1
Kroes 2011 [49]	Netherlands	1	1			1/1	1/1	ND	1/1	1/1
Neill 2011 [50]	USA	1	1			1/1	ND	ND	ND	ND
Mayo 2011 [13]	Spain	1		1		1/1	0/1	1/1	ND	ND
Budisteanu 2011 [51]	Romania	1	1			1/1	1/1	0/1	1/1	1/1
Grasshoff 2011 [14]	Germany	2		2		2/2	ND	0/2	1/2	1/1
Breman 2011 [52]	USA	6	6			6/6	6/6	0/6	5/6	ND
Jezela-Stanek 2011 [53]	Poland	1	1			1/1	1/1	1/1	1/1	1/1
Carvalho 2011^a^ [54]	USA	7	7			7/7	7/7	2/7	6/6	6/6
Honda 2012 [55]	Japan	12	12			12/12	10/12	9/12	10/12	10/10
Honda 2012 [56]	Japan	2	2		1	2/2	2/2	2/2	2/2	1/1
Van Esch 2012 [57]	Belgium	15	15			13/13	14/14	8/14	9/13	ND
Bijlsma 2012^a^ [15]	USA	4		4		3/4	2/4	0/4	3/4	2/3
De Palma 2012 [58]	Italy	1	1			1/1	ND	1/1	1/1	1/1
Vignoli 2012 [59]	Italy	8	8			8/8	5/5	6/8	6/6	6/6
Sanmann 2012 [60]	USA	6	6		4	6/6	3/3	2/2	5/5	5/5
Xu 2012 [61]	China	2	2			2/2	2/2	0/2	0/2	ND
Utine 2012 [62]	Turkey	1	1			1/1	1/1	0/1	1/1	1/1
Tang 2012 [63]	UK	2	2			3/3	3/3	2/2	3/3	3/3
Lund 2013 [64]	Norway	1	1			1/1	1/1	1/1	1/1	1/1
Peters 2013^c^ [65]	USA	10	10			ND	9/10	ND	10/10	ND
Peters 2013^c^ [66]	USA	6	6			ND	ND	ND	ND	ND
Shimada 2013 [67]	Japan	4	3	1		4/4	3/3	3/4	4/4	3/3
Shimada 2013 [16]	Japan	3	2	1		3/3	3/3	3/3	3/3	3/3
Yamamoto 2014^a^ [68]	Japan	4	4			4/4	2/2	3/4	1/1	ND
Caumes 2014 [69]	France	8	8			8/8	8/8	8/8	3/8	ND
Scott Schwoerer 2014 [17]	USA	2		2		2/2	2/2	2/2	1/2	1/1
Fukushi 2014 [70]	Japan	5	5			5/5	4/4	5/5	5/5	4/4
Fieremans 2014 [18]	Belgium	2		2		2/2	ND	0/1	ND	ND
Nascimento 2014 [71]	Canada	1	1			1/1	ND	1/1	ND	ND
Yi 2014^d^ [72]	China									
Wang 2014^d^ [73]	China									
Novara 2014 [19]	Italy	3		3	2	3/3	3/3	ND	0/3	ND
Lin 2014 [74]	Taiwan	1	1			1/1	1/1	1/1	1/1	1/1
Chow 2015 [75]	Singapore	3	3			3/3	3/3	3/3	3/3	3/3
Bauer 2015 [76]	Germany	12	12			ND	ND	ND	10/10	9/10
Zhang 2015 [77]	China	1	1		2	1/1	1/1	0/1	1/1	1/1
Voinova 2015 [78]	Russia	4	4			4/4	4/4	2/4	4/4	ND
Magini 2015^b^ [79]	Italy									
Yi 2016 [80]	China	16	15	1		16/16	16/16	6/16	16/16	ND
El Chehadeh 2016^c^ [81]	France					ND	ND	15/30	ND	ND
Ha 2016 [82]	USA	1	1		1	1/1	1/1	0/1	ND	ND
Tug 2016 [83]	Turkey	1	1			1/1	1/1	0/1	1/1	ND
Trobaugh‐Lotrario 2016 [84]	USA	1	1			1/1	ND	1/1	1/1	1/1
San Antonio-Arce 2016 [20]	Spain	2		2		2/2	1/2	1/2	2/2	2/2
Lim 2017 [85]	Australia	56	49	7		45/55	35/55	24/45	41/56	41/41
El Chehadeh 2017 [21]	France	4		4		4/4	3/4	2/4	3/4	0/2
Moirangthem 2017 [86]	India	2	2		3	2/2	1/1	0/2	1/2	1/1
Tsuji-Hosokawa 2017 [87]	Japan	1	1			1/1	ND	0/1	1/1	1/1
Yon 2017 [88]	Korea	2	2			2/2	2/2	2/2	2/2	2/2
Tang 2017^d^ [89]	China	3	3			2/2	3/3	2/2	3/3	ND
Li 2017 [90]	China	5	5		1	5/5	5/5	4/5	4/5	4/4
Deshwar 2018 [91]	Canada	1	1			1/1	1/1	ND	1/1	1/1
Miguet 2018 [92]	France	86	86			59/59	57/58	35/59	49/55	49/49
Giudice-Nairn 2019^c^ [93]	Australia	20	16	4		19/20	15/20	11/20	18/20	15/18
Jiang 2019^d^ [94]	China									
Peters 2019 [95]	USA	48	43	5		31/48	42/48	21/48	27/48	27/27
Hirabayashi 2019 [96]	Japan	1	1			1/1	1/1	1/1	1/1	ND
Pascual-Alonso 2020^a^ [97]	Spain	19	18	1		19/19	17/19	19/19	14/18	14/14
Choi 2020 [98]	Hong Kong	1	1			1/1	1/1	0/1	1/1	1/1
Tekendo-Ngongang 2020 [99]	Cameroon	1	1		1	1/1	1/1	1/1	1/1	1/1
van Baelen 2020 [100]	Belgium	3	3			2/2	2/2	ND	3/3	3/3
Liu 2020^d^ [101]	China									
Gutierrez-Sanchez 2020^c^ [102]	Spain	2	2			2/2	1/1	ND	2/2	1/1
Takeguchi 2021 [103]	Japan	24	24			18/18	18/21	16/24	18/23	14/18
		545	504	41	39	462/491	411/454	257/459	376/479	316/324
			92%	8%		94%	91%	56%	78%	98%

^a^Part of cohort not included in calculation as published elsewhere

^b^Whole cohort was published elsewhere

^c^Not included in calculation a possibly published elsewhere

^d^Article available in Chinese only

Seventy-eight percent of reported patients (376/479) suffer from recurrent or severe infections (Table 1). Most common are respiratory infections with 98% of reported cases (316/324), but patients also present with otitis media, urinary tract infections, and sepsis (Table 1). Early death (defined as < 25 years) is reported with a frequency of 4 to 55% [1, 57, 92, 95]. Among the 67 patients with described cause of early death, 58 (87%) died in the context of a severe infection at the age of 3 weeks to 24 years (median 11 years; data available for 43 patients only) [1, 2, 5–7, 9, 12, 25, 26, 32, 33, 38, 39, 43, 54–57, 63, 67, 70, 75, 76, 80, 84, 86, 87, 90, 92, 93, 95, 97, 99, 104]. Eighty-two percent (328/398) of males suffer from recurrent or severe infections but only 61% (20/33) of the described females. Few studies further examined the detailed infectious and the underlying immunological phenotype of the patients. In contrast to the widespread notion of “recurrent severe infections,” information about identified pathogens is only available for 19 patients [7, 12, 16, 54, 76, 100, 105]. Among the 55 isolated pathogens were 45 bacteria (most of all *S. pneumoniae*, *H. influenzae*, *E. coli*, and *S. aureus*), 6 viruses, and 4 *Candida* (Table S1). However, as the total viable counts are not stated, it remains unclear if these were the disease-causing pathogens. Bronchoalveolar lavage for the identification of pathogens was only performed in 7 patients [76, 105].

Few studies have examined patients for their immunological phenotype [33, 39, 76, 88, 100]. The most common characteristic is a poor response to vaccination especially against *Streptococcus pneumoniae* which was described in 15/26 patients [33, 39, 76]. Some patients show selective deficiency of immunoglobulin (Ig) A (11/47) and/or IgG2 (7/24) [23, 26, 30, 33, 39, 46, 56, 63, 75, 76, 88, 100, 104]. Moreover, several patients present with episodes of unexplained fever and remarkably high C-reactive protein (CRP) values during non-invasive infections [24, 43, 54, 70, 76, 105]. In 2015, Bauer et al. suggested the substitution of polyvalent IgG in patients with an IgG2 subclass deficiency and/or low post-vaccination titers against pneumococci who suffer from recurrent infections—eventually combined with prophylactic antibiotics [76, 105]. In the 26 studies published since 2016, only three evaluated the immunoglobulin levels, and none mentioned the response to vaccination [88, 100, 103]. Four patients were mentioned to receive antibiotic prophylaxis [93, 100, 105]. As infections still limit the quality of life and are the most common cause of death in MDS patients, there seems to be an unmet clinical need regarding pneumological and microbiological diagnostics as well as targeted anti-infectious treatment [92].

**Fig. 1:**
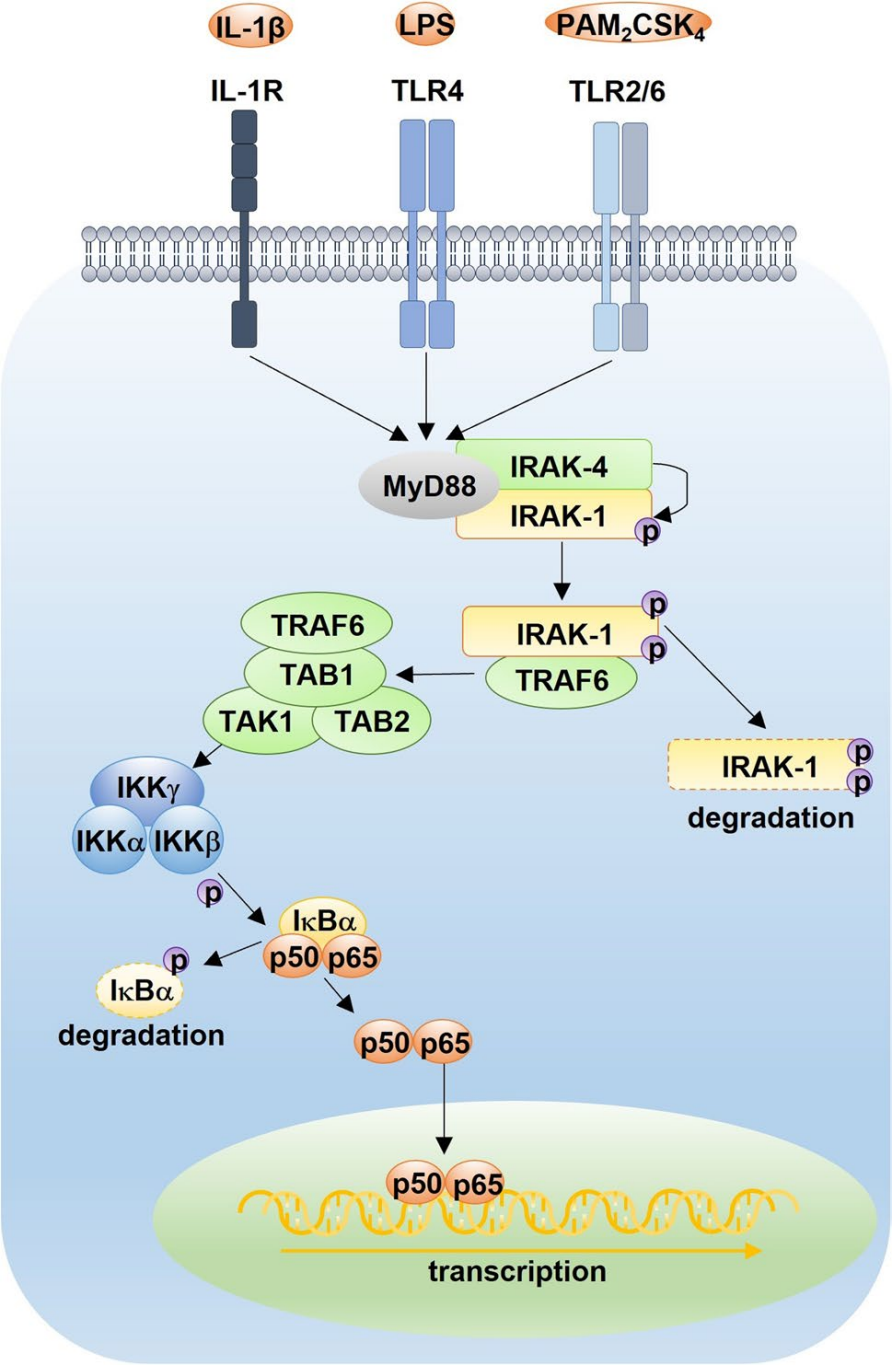
Canonical pathway of NF-κB signaling. Upon binding of ligands, such as IL-1 to the IL-1R, LPS to TLR4, or diacylated lipopeptides like PAM2CSK4 to TLR2/6, the inflammatory signaling is mediated via the myddosome complex which consists of MyD88 and IRAK family kinases. MyD88 associates with IRAK4 via a homophilic interaction between their death domains. IRAK4 induces the phosphorylation of IRAK1. The hyperphosphorylated IRAK1 then dissociates from the complex and associates with TRAF6 to activate TAK-1/TAB complex. The latter enhances the activity of the IKK complex which then leads to phosphorylation and degradation of IκB. Thereby, NF-κB dimers such as p65 (RelA) and p50 are activated and migrate into the nucleus which results in gene transcription and the induction of inflammatory cytokines. (Adapted from Heller S, Kölsch U, Magg T, et al. T Cell Impairment Is Predictive for a Severe Clinical Course in NEMO Deficiency. J Clin Immunol. 2020;40(3):421–434. Used with permission.) IκBα, NF-κB inhibitor α; IKK, IκB kinase; IL-1R, interleukin-1 receptor; IRAK, interleukin-1 receptor–associated kinase; LPS, lipopolysaccharide; MyD88, myeloid differentiation primary response 88; TAB, TAK1-binding proteins; TAK-1, TGF-β–activated kinase 1; TLR, toll-like receptor; TRAF, TNF receptor–associated factor. The canonical NF-κB pathway can be disturbed through disease-causing mutations within multiple genes. Described correlations between phenotype and genotype are listed in the OMIM database (OMIM numbers: IκBα*164,008, IKKα*600,664, IKKβ*603,258, IKKγ*300,248, IRAK1*300,283, IRAK4*606,883, MyD88*602,170, p50*164,011, p65*164,014)

It remains unknown whether recurrent fever and strong acute phase response in these patients are rather driven by infections which are difficult to clear and/or by autoinflammation. Throughout the manuscript, we use the term autoinflammation which describes systemic inflammatory processes due to a non-infectious (auto)activation of the innate immune system. Both hypotheses, the one of an “infectious fever” and the one of an “autoinflammatory fever,” are not mutually exclusive [31, 104, 106, 107]. In 2009, Kirk et al. suspected a link between *IRAK1* duplication and susceptibility to infection [43]. IRAK1 participates in multiple IL-1 and TLR–driven signaling processes that regulate immunity and inflammation [108–114]. For instance, IRAK1 plays an important role in the regulation of both, the interleukin-1 (IL-1)–mediated and the Toll-like receptor (TLR)–mediated, so-called canonical signaling pathways of NF-κB (nuclear factor “kappa-light-chain-enhancer” of activated B cells) (Fig. 1). Upon binding, IL-1 receptors with their respective cytokine or TLR with their respective ligand recruit the adaptor protein myeloid differentiation primary response 88 (MyD88) which associates with IRAK4 via a homophilic interaction between their death domains. IRAK4 induces the phosphorylation of IRAK1. The hyperphosphorylated IRAK1 then dissociates from the complex and associates with TNF receptor–associated factor 6 (TRAF6) to activate TAK-1/TAB (TGF-β–activated kinase/TAK1-binding proteins). The latter enhances the activity of the IκB kinase (IKK) complex, which in turn leads to phosphorylation and degradation of inhibitors of nuclear factor kappa B (IκB). Thereby, NF-κB dimers comprising p65 (RelA), c-Rel, and p50 are activated and migrate into the nucleus which results in gene transcription and the induction of inflammatory cytokines such as tumor necrosis factor α (TNF-α), IL-1β, IL-6, and IL-12 [108–114].

Della Mina et al. examined the canonical NF-κB signaling in an *IRAK1*-null patient [115]. The patient’s fibroblasts showed poor responses upon stimulation with TLR2/6 and TLR4 agonists but unimpaired responses to IL-1β. The patient’s peripheral blood mononuclear cells (PBMCs) responded normally to IL-1β as well as TLR2/6 and TLR4 agonists [115]. Responses to TLR3 agonist Poly(I:C) were not influenced as it signals via TRIF-dependent pathways [115].

The combination of the clinical phenotype in MDS and the duplication of the *IRAK1* gene brings up the question if IRAK1 overexpression causes increased canonical NF-κB signaling and detrimentally increased acute phase responses. Considering the results of Della Mina et al., we hypothesized that patients with *MECP2/IRAK1* duplication might show enhanced cytokine production in fibroblasts upon simulation with TLR2/6 and TLR4 agonists. Therefore, we evaluated the production of proinflammatory cytokines as well as the IκBα degradation and IRAK1 phosphorylation upon stimulation with IL-1β and TLR agonists in SV40-immortalized fibroblasts of 9 patients with *MECP2/IRAK1* duplication syndrome, respectively. Additionally, we investigated the production of proinflammatory cytokines not only in PBMCs but also whole blood.

## Methods

### Patients

The study was conducted in accordance with the ethical standards of the 1964 Helsinki declaration and the institutional research committee (Charité-Universitätsmedizin Berlin, Germany, EA2/063/12). Informed consent was obtained from each patient or the patients’ parents. Our cohort consists of 9 male patients diagnosed with MDS. We recruited them by contacting patients who participated as well as physicians who cooperated in our previous study [76]. Five of the patients were described before [76, 105, 116]. A duplication of at least *MECP2* and *IRAK1* was confirmed in all patients enrolled by array-based Comparative Genomic Hybridization (array-CGH) prior to this study. We standardized the ranges of the duplications to Genome Reference Consortium Human Build 37 (GRCh37) by the NCBI Genome Remapping Service to compare the duplication size of all patients.

### Material

Fibroblasts of P1, P2, P3, and P4 as well as of 4 healthy individuals were obtained by skin biopsies and immortalized by simian virus (SV40) as described previously [76, 117]. Blood samples of P3, P5, P6, P7, P8, and P9 as well as of healthy controls were acquired in parallel to routine blood tests. As P1 and P2 deceased, and we were not able to contact P4 recently, we were not able to obtain current blood samples from P1, P2, and P4. P5-9 did not donate fibroblasts. We isolated the PBMCs and performed the analysis in our laboratory with the same methods and equipment.

### Cell Stimulation and Cytokine Determination (ELISA)

Levels of IL-6 and IL-8 production were assessed in SV40-fibroblasts of P1, P2, P3, and P4 as well as of healthy controls and IRAK1-deficient and IRAK4-deficient controls incubated for 24 h in the presence of IL-1β (1 ng/ml, R&D Systems), TNF-α (20 ng/ml, R&D Systems), Phorbol-12-Myristat-13-Acetat (PMA)/Ionomycin (1 × 10^−7^ M/1 × 10^−5^ M, Sigma-Aldrich), or TLR agonists (TLR4 agonist LPS (10 µg/ml, Sigma-Aldrich), TLR2/6 agonist PAM_2_CSK_4_ (10 µg/ml, Invivogen), or TLR3 agonist Poly(I:C) (25 µg /ml, Invivogen)), as well as in PBMCs of P3, P5, P6, P7, P8, and P9 as well as of healthy controls incubated for 48 h in the presence of IL-1β (1 ng/ml, R&D Systems), TNF-α (20 ng/ml, R&D Systems), PMA/Ionomycin (1 × 10^−7^ M/1 × 10^−5^ M, Sigma-Aldrich), or TLR agonists (TLR4 agonist LPS (1 ng/ml, Sigma-Aldrich) or TLR2/6 agonist PAM_2_CSK_4_ (1 µg/ml, Invivogen)). Cytokine concentrations in cell culture supernatants were assessed by enzyme-linked immunosorbent assay (ELISA) using “PeliPair reagent” sets (Sanquin) for human IL-6 and IL-8 according to the manufacturer’s protocol. The experiment was conducted three times.

IL-6 and IL-10 levels were measured in heparinized whole blood of P3, P5, P6, P7, P8, and P9 incubated for 48 h in the presence of IL-1β (20 ng/ml, R&D Systems), TNF-α (20 ng/ml, R&D Systems), PMA/Ionomycin (1 × 10^−7^ M/1 × 10^−5^ M, Sigma-Aldrich), or TLR agonists (TLR4 agonist LPS (1 ng/ml, Sigma-Aldrich) or TLR2/6 agonist PAM_2_CSK_4_ (100 ng/ml, Invivogen)). The cytokine concentrations were measured by ECLIA by Labor Berlin on an IMMULITE® 1000 (Siemens) and compared to a cohort of healthy controls assessed in our laboratory (*n* = 179) [118].

### Western Blots

To analyze IRAK1 expression and IκBα degradation, we stimulated SV40-immortalized fibroblasts of P1, P2, P3, and P4 as well as of healthy controls and IRAK1-deficient and IRAK4-deficient controls with IL-1β (10 ng/ml, R&D Systems) and TNF-α (20 ng/ml, R&D Systems) for 15, 30, 45, 60, and 90 min as well as with IL-1β (10 ng/ml, R&D Systems), TLR4 agonist lipopolysaccharide (LPS) (10 µg/ml, Sigma-Aldrich), and TNF-α (20 ng/ml, R&D Systems) for 20, 60, 120, and 240 min, respectively. The further steps were performed as described previously using the following antibodies: IκBa (610,690, BD Biosciences), IRAK1 (sc-7883, Santa Cruz Biotechnology), IRAK-4 (ADI-KAP-ST206-E, Enzo), glycerinaldehyd-3-phosphat-dehydrogenase (GAPDH) (sc-25778, Santa Cruz Biotechnology), Goat Anti Rabbit IgG (111–035-045, Dianova), and Goat Anti Mouse IgG (115–035-062, Dianova) [118]. Detailed protocols are available upon request.

### Graphs and Statistical Information

Graphs were created using GraphPad Prism 9 software (GraphPad Software Inc.) and PowerPoint (Microsoft Office). Statistical analyses were performed using SPSS V28.0.1.0 (IBM). Data sets were tested for normal distribution, and statistical comparisons were done using a Mann–Whitney *U* test. For comparison of multiple groups, Kruskal–Wallis test was used. *P* values of less than 0.05 after adjusting by Bonferroni method were considered significant. *P < 0.05, **P < 0.01, ***P < 0.001, ****P < 0.0001.

## Results

### Patients with *MECP2/IRAK1* Duplication Suffer from Recurrent Respiratory Infections

**Fig. 2:**
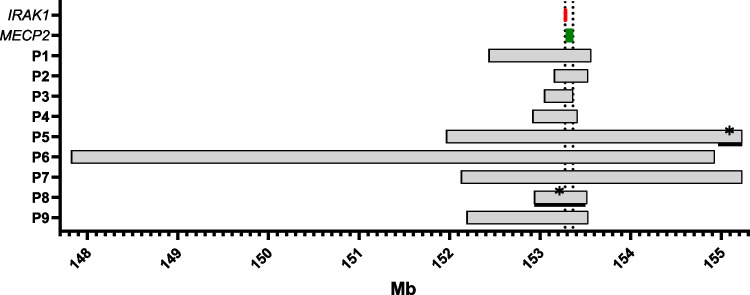
Size of duplications on chromosome Xq28 based on human genome assembly GRCh37 (hg19) in mega base pairs (Mb). Position of *IRAK1* is indicated in red and position of *MECP2* is indicated in green. Vertical dotted lines show the minimal duplicated region including *IRAK1* and *MECP2*. *Black bars with a star indicate triplicated segments. This figure was created using Prism 9 (GraphPad). The exact boundaries and the included genes are shown in the Supplementary Information

Our 9 patients show duplications of variable sizes at least encompassing the neighboring genes *MECP2* and *IRAK1* (Fig. 2). In patients 5 and 8, part of the region is triplicated. The exact boundaries and the included genes are shown in the Supplementary Information. All patients suffered from recurrent or severe infections, mostly respiratory infections, which often required hospitalization.

Patient 3 is a 25-year-old patient who is followed-up in our department at least 4 times a year and was clinically and molecularly characterized before [P1 in 76, 105]. Array comparative genome hybridization (array-CGH) confirmed a duplication of 1.1 Mb at Xq28. He first presented with global developmental delay, muscular hypotonia, and spastic tetra paresis. He suffers from epilepsy and recurrent severe infections. Of his in total 64 episodes of pneumonia, he had developed 47 until his 14th y/a. After starting an immunoglobulin substitution (at 12 y/a) as well as supportive measures and antibiotic prophylaxis (at 14 y/a), the frequency of infections declined, leading to 3 episodes of pneumonia only in the following 8 years. Despite this treatment, we recorded an increase of hospital admissions due to infections in the last 2 years including 12 episodes of pneumonia and 2 episodes of sepsis (Fig. S1). Throughout the last years, the boy developed chronic aspiration and shows bronchiectasis in his latest CT scans (Fig. S2). We are now detecting opportunistic pathogens such as a multidrug-resistant *Citrobacter freundii* as well as *Candida glabrata* and *Trichosporon asahii* in bronchoalveolar lavages. He is currently under prophylactic anti-infective treatment with cotrimoxazole, penicillin, and fluconazole. In all infectious episodes, our patients presented with fever above 39 °C and high CRP levels, typically above > 100 mg/dl, already during the first 3 days of the infection. The boy shows a normal total immunoglobulin titer but deficiency of IgG2, IgG4, IgA, and IgM. A polysaccharide-specific antibody deficiency persisted despite repeated vaccinations.

The baseline clinical features of all patients in our cohort are summarized in Table 2. Detailed case reports of P1, P2, and P4–P9 are provided in the Supplementary Information.

**Table 2:** Clinical baseline characteristics of the cohort. UTI, urinary tract infections; SV40, SV40-immortalized fibroblasts; PBMCs, peripheral blood mononuclear cells; ND, not determined; x, present; /, absent

Patient	Sex	Age at last consultation	Origin	Intellectual disability or developmental delay	Infantile hypotonia	Epileptic seizures	Recurrent or severe infections	Number of				Material
								Pneumonia	Sepsis	UTI	Purulent otitis	
^1a^	Male	15†	Germany	x	x	x	x	14	0	7	2	SV40
2^a^	Male	15†	Belgium	x	x	x	x	50	1	ND	3	SV40
3^a^	Male	25	Germany	x	x	x	x	58	> 5	> 1	> 3	SV40, PBMCs, whole blood
4^a^	Male	8	France	x	x	ND	x	2	0	ND	> 4	SV40
5	Male	21	Belgium	x	x	x	x	32	1	1	ND	PBMCs, whole blood
6	Male	10	Belgium	x	x	x	x	3	0	0	ND	PBMCs, whole blood
7	Male	5	Poland	x	x	/	x	5	1	2	ND	PBMCs, whole blood
8	Male	10	Germany	x	x	/	x	> 1	0	ND	ND	PBMCs, whole blood
9^a^	Male	10	Germany	x	x	ND	x	7	ND	5	1	PBMCs, whole blood
				9/9	9/9	5/7	9/9					

^a^Published elsewhere, see Supplementary Information

^†^Deceased

### *IRAK1* Duplication Leads to Increased Protein Levels in Patient-Derived Fibroblast Cell Lines

**Fig. 3:**
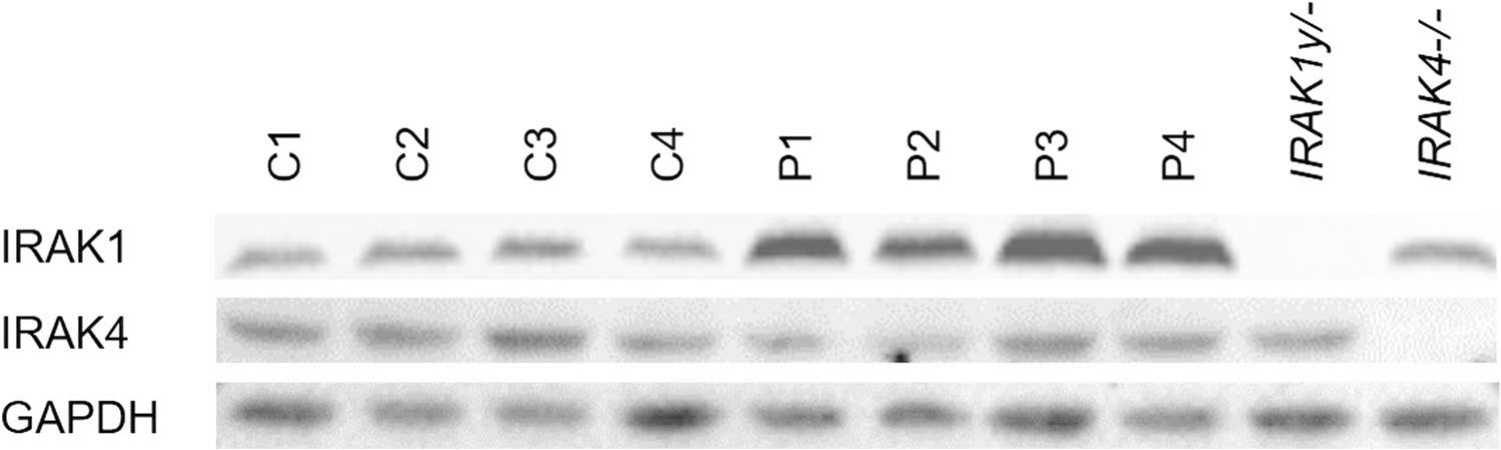
Characterization of SV40-immortalized fibroblasts. Western blot analysis of IRAK-1 and IRAK-4 protein levels in cell lysates from SV40-immortalized fibroblasts of healthy controls (*n* = 4), patients (*n* = 4), an IRAK1-deficient patient, and an IRAK4-deficient patient. Pictures were cropped and adjusted. Quantitation is shown in Table S2

First, we characterized the SV40-immortalized fibroblasts cell lines of both patients and healthy controls for their expression of IRAK1, IRAK4, and GAPDH (Fig. 3). We used an IRAK1-deficient and an IRAK4-deficient cell line as negative controls. The patients’ cells (P1–P4) contained at least twice as much IRAK1 as the cells of the healthy controls (C1–C4) (Fig. 3). The calculated ratios are stated in the Supplementary Information.

### *IRAK1* Duplication Does Promote Excessive Cytokine Production Neither in Fibroblasts Nor in PBMC Nor in Whole Blood

We hypothesized that the susceptibility to infection could be caused by a hyperinflammatory immune response due to increased canonical NF-κB signaling because of IRAK1 overexpression. Therefore, we determined the impact of the *MECP2* and *IRAK1* duplication on the canonical NF-κB signaling. Hence, we performed an ELISA to measure the cytokine production in the cell culture supernatants of SV40-immortalized fibroblasts, PBMCs, and whole blood upon stimulation with IL-1β as well as the TLR agonists LPS (TLR4), PAM_2_CSK_4_ (TLR2/6), and Poly(I:C) (TLR3). We used TNF-α and PMA/Ionomycin as NF-κB–independent intra-assay controls.

**Fig. 4:**
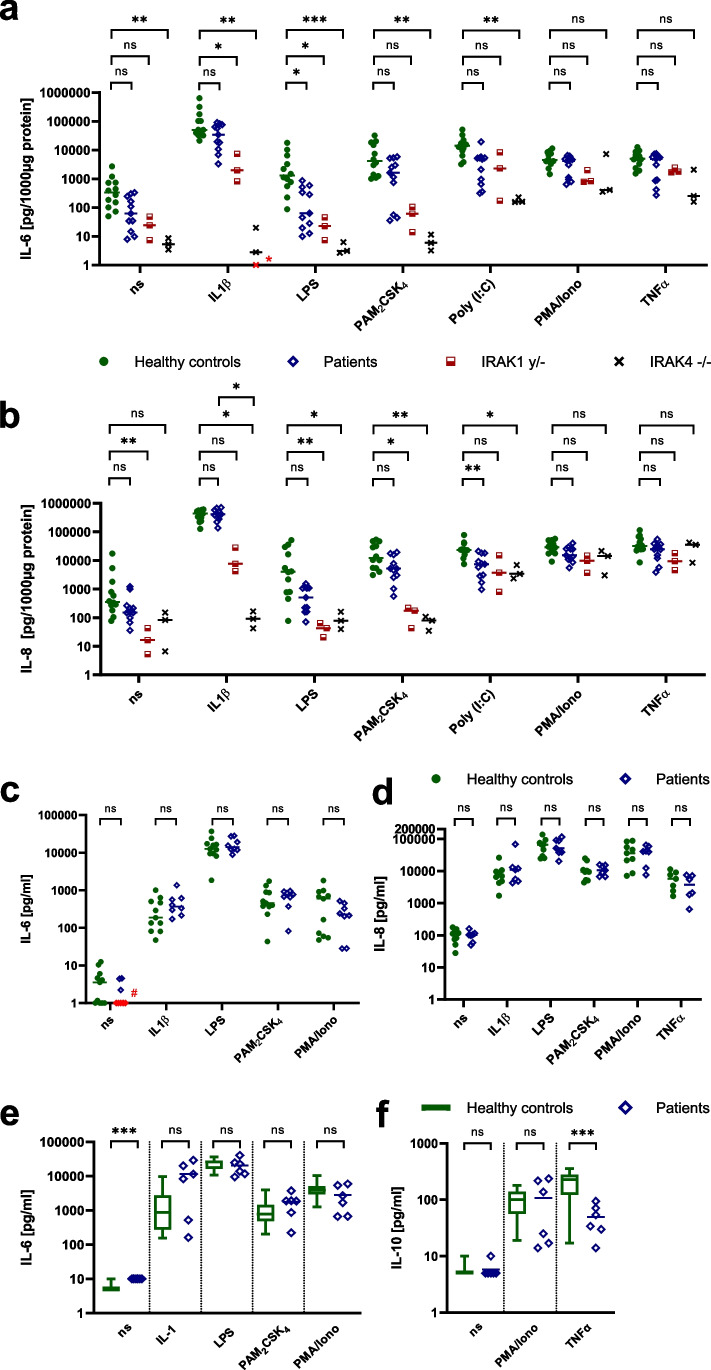
Cytokine production upon stimulation measured in cell culture supernatants. Bars indicate median values. **a** and **b** Cytokine production in patients’ SV40-immortalized fibroblasts (*n* = 4) upon stimulation with IL-1β (1 ng/ml), TLR agonists (LPS for TLR4 (10 µg/ml), PAM_2_CSK_4_ for TLR2/TLR6 (10 µg/ml), Poly(I:C) for TLR3 (25 µg/ml)), PMA/Iono (1 × 10^−7^ M/1 × 10^−5^ M), and TNF-α (20 ng/ml). SV40-immortalized fibroblasts of healthy controls (*n* = 4), an *IRAK1*-null patient, and an *IRAK4*-null patient were used as intra-assay controls. The experiment was conducted three times. **a** IL-6 production. **b** IL-8 production. **c** and **d** Cytokine production in patients’ PBMCs upon stimulation with IL-1β (1 ng/ml), TLR agonists (LPS for TLR4 (1 ng/ml), PAM_2_CSK_4_ for TLR2/TLR6 (1 µg/ml)), PMA/Iono (1 × 10^−7^ M/1 × 10^−5^ M), and TNF-α (20 ng/ml). PBMCs of healthy controls were used as intra-assay controls. **c** IL-6 production (*n* = 8 for patients and *n* = 11 for healthy controls). **d** IL-8 production (*n* = 7 for patients and *n* = 9 for healthy controls). # zero values. **e** and** f** Cytokine production in patients’ whole blood (*n* = 6) upon stimulation with IL-1β (20 ng/ml), TLR agonists (LPS for TLR4 (1 ng/ml), PAM_2_CSK_4_ for TLR2/TLR6 (100 ng/ml)), PMA/Iono (1 × 10^−7^ M/1 × 10^−5^ M), and TNF-α (20 ng/ml). The analyses were performed in comparison to a cohort of healthy controls assessed in our laboratory (*n* = 179, whiskers 5–95 percentile). **e** IL-6 production. **f** IL-10 production. This figure was created using Prism 9 (GraphPad). *P < 0.05, **P < 0.01, ***P < 0.001, ****P < 0.0001, ns, not significant. If there was no statistically significant difference (patients vs. *IRAK1y/-*; patients vs. *IRAK4-/-* and *IRAK1y/-* vs. *IRAK4-/-*), *P* values were not plotted (Fig. 4a and 4b). IRAK1, IL-1 receptor–associated kinase 1; IL-1β, interleukin-1β; LPS, lipopolysaccharide; PAM_2_, PAM_2_CSK_4_; PMA/I, phorbol myristate acetate/ionomycin; TLR, toll-like receptor; TNF-α, tumor necrosis factor α

Production of IL-6 and IL-8 upon stimulation with IL-1β or TLR2/6 agonist PAM_2_CSK_4_ was increased in fibroblasts of both healthy controls and *MECP2/IRAK1*-duplicated patients, but we did not see a difference between the two groups (Fig. 4a and 4b). Interestingly and in contrast to our hypothesis, the data suggests that the cytokine production upon stimulation with TLR4 agonist (LPS) in immortalized fibroblasts of *MECP2/IRAK1*-duplicated patients is lower than in healthy controls. In IRAK4-deficient fibroblasts, cytokine production upon stimulation with IL-1β, TLR4 agonist LPS, and TLR2/6 agonist PAM_2_CSK_4_ was absent (Fig. 4a and 4b). In IRAK1-deficient fibroblasts, we found no response to TLR4 agonist LPS and TLR2/6 agonist PAM_2_CSK_4_ but an almost unimpaired response to IL-1β (Fig. 4a and 4b). All fibroblasts showed increased cytokine production upon activation with TLR3 agonist Poly(I:C), TNF-α, and PMA/Ionomycin (Fig. 4a and 4b). The response upon stimulation with IL-1β, TLR agonists, and TNF-α was similar for PBMCs and whole blood in both groups (Fig. 4c–f). *P* values for significant differences are indicated in Fig. 4. If there was no statistically significant difference (patients vs. *IRAK1y/-*; patients vs. *IRAK4-/-* and *IRAK1y/-* vs. *IRAK4-/-*), *P* values were not plotted (Fig. 4a and 4b).

These results indicate that *MECP2/IRAK1* duplication does not lead to a higher amount of inflammatory cytokines upon stimulation in immortalized fibroblasts, PBMCs, and whole blood.

### Normal IRAK1 and IκBα Degradation Indicates Regular NF-κB Signaling in Patient-Derived Fibroblasts with *IRAK1* Duplication

**Fig. 5:**
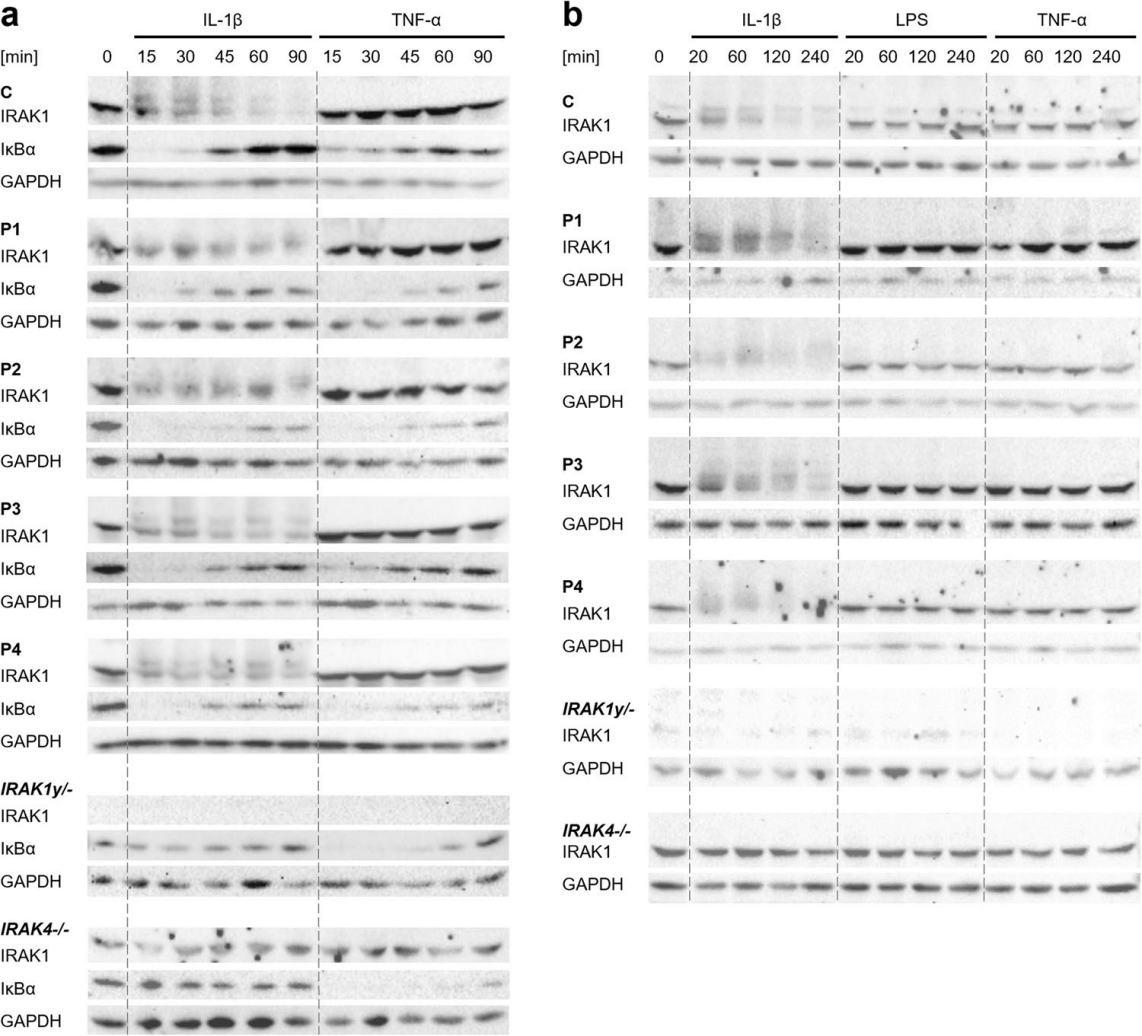
Immunological phenotype of SV40-immortalized fibroblasts. **a** IRAK1 phosphorylation and IκBα degradation upon stimulation with IL-1β (10 ng/ml) and TNF-α (20 ng/ml). **b** IRAK1 phosphorylation upon stimulation with IL-1β (10 ng/ml), TLR4 agonist LPS (10 µg/ml), and TNF-α (20 ng/ml). Cell lysates were analyzed by performing Western blots specific to IRAK1, IκBα, and GAPDH. Phosphorylated IRAK1 forms a smear above the band of the non-phosphorylated IRAK1. GAPDH was used as a loading control. Pictures were cropped and adjusted. IRAK1, IL-1 receptor–associated kinase 1; IκBα, NF-κB inhibitor α; GAPDH, glyceraldehyde-3-phosphate-dehydrogenase; IL-1β, interleukin-1β; LPS, lipopolysaccharide; TNF-α, tumor necrosis factor α

Having observed that *MECP2/IRAK1* duplication does not cause an increased acute inflammatory response in vitro, we hypothesized that canonical NF-κB signaling may be altered regarding the phosphorylation and degradation of involved proteins such as IRAK1 and IκBα. To further delineate this pathway, we performed Western blots on whole cell lysates of SV40 fibroblasts of P1–P4, a healthy control (C), as well as IRAK1- and IRAK4-deficient controls stimulated with IL-1β as well as TNF-α as an NF-κB–independent intra-assay control (Fig. 5a). Upon activation, IRAK1 is phosphorylated leading to a higher mass of the molecule. Phosphorylated IRAK1 becomes hence visible as a smear above the band of non-phosphorylated IRAK1. It appears as if there is still non-phosphorylated IRAK1 left 90 min after stimulation with IL-1β (Fig. 5a). In a second experiment, we assessed the IRAK1 phosphorylation upon IL1β and TNF-α for longer time frames up to 240 min and additionally stimulated the SV40 fibroblasts with TLR4 agonist LPS (Fig. 5b). Both, the patients’ and the control’s cells showed a similar pattern of IRAK1 phosphorylation and degradation upon activation with IL-1β, whereas it was absent in IRAK4-deficient fibroblasts (Fig. 5a and 5b). Self-explanatory, the IRAK1-deficient fibroblasts did not contain any IRAK1 protein, and therefore, it could not be phosphorylated or degraded (Fig. 5a and 5b). IRAK1 phosphorylation and degradation could not be induced by LPS or TNF-α stimulation in any of the cell lysates (Fig. 5a and 5b). The time pattern of IκBα degradation upon IL-1β stimulation and the reoccurrence of non-phosphorylated IκBα was similar in cells of the patients and the control tested, whereas it was absent in IRAK1*-* and IRAK4-deficient fibroblasts (Fig. 5a). Overall, our data suggests that there is no difference in the activity of the canonical NF-κB signaling in *MECP2/IRAK1*-duplicated patients.

## Discussion

Although research on *MECP2/IRAK1* duplication syndrome has increased, a comprehensive pathophysiological mechanism that explains the frequency and severity of infections, the most common cause of death, remains unknown. Numerous publications describe patients who repeatedly require hospitalization, invasive ventilation, and intensive care admission [63, 75, 76, 103, 105]. In P3, pneumococcal immunization and antibiotic prophylaxis reduced the number of infections per year drastically for many years (Fig. S1). However, after successful long-term prophylaxis on antibiotics and IgG, he has been presenting multiple times with pneumonia caused by multidrug-resistant and rare pathogens since the age of 23. Patients like these show that the control of infections clearly is still an unmet clinical need.

IRAK1 participates in multiple IL-1 and TLR–driven signaling processes that regulate immunity and inflammation [108–113]. Therefore, we hypothesized that the infections may be triggered by a strong acute phase response due to IRAK1 overexpression and subsequently increased canonical NF-κB signaling. However, in our cohort, we did not see any evidence of increased IRAK1-dependent degradation of IκBα. We demonstrated that the production of proinflammatory cytokines IL-6 and IL-8 upon stimulation with IL-1β and TLR2/6 agonist PAM_2_CSK_4_ is similar in immortalized fibroblasts as well as PBMCs and whole blood of patients with *MECP2/IRAK1* duplication and healthy controls. Also, we did not see an enhanced response upon stimulation with TLR4 agonist LPS in PBMCs and whole blood of patients compared to healthy controls. The results in our healthy controls as well as our IRAK1- and IRAK4-deficient controls were similar to the results of Della Mina et al. [115]. Response to IL-1β and TLR agonists seems to be normal not only in PBMCs but also whole blood which suggests that canonical NF-κB signaling is also neither increased nor impaired in neutrophilic granulocytes of patients with *MECP2/IRAK1* duplication.

This raises the question of whether the inflammation (as documented by high CRP levels in many patients) observed in MDS patients might rather be driven by an infectious than an autoinflammatory process. Ninety-one percent (411/454) of the published patients suffered from muscle hypotonia suggesting that an insufficient occlusion of the gastric sphincter, as well as swallowing difficulties, could favor aspiration (Table 1). Gastroesophageal reflux with subsequent aspiration was suggested as a cause for frequent respiratory tract infections [31, 104]. However, only 55% (149/270) of the patients were described to suffer from reflux, whereas 78% of the patients (376/479) were described to suffer from recurrent or severe infections ([3, 5, 6, 9, 15, 18, 26, 27, 31, 33, 38, 52, 54–56, 60, 67, 75, 84, 85, 92, 93, 95, 97, 98, 100, 103, 104, 119], Table 1).

The NF-κB signaling in fibroblasts and blood seems to be unimpaired. However, this might be different in other tissues such as lung epithelia. On the one hand, IRAK-1 was shown to be essential for IL-8 production in human airway epithelial cells [120]. On the other hand, IRAK-1 is necessary for the rhinovirus-stimulated induction of CXCL-10 in airway epithelial cells and macrophages [121]. Both excessive production of IL-8 and CXCL-10 could contribute to lung inflammation leading to the clinical phenotype of MDS patients. From a scientific point of view, it seems interesting to study the cytokine production and CXCL-10 induction in airway epithelial cells of patients with *MECP2/IRAK1* duplication. However, it seems almost impossible to obtain sufficient amounts of primary lung tissue from children with such a rare disease in a standardized way, let alone enough to culture lung epithelia. An alternative strategy to investigate the role of IRAK1 in lung epithelia might be to differentiate human-induced pluripotent stem cells (hiPSCs) to lung epithelial cells [122].

Yang et al. proposed that severe infections in MDS patients occur due to the lack of TH1 response and subsequently low IFN-γ activity [106]. However, a generally impaired IFN-γ secretion could not be reproduced by Bauer et al. [76]. Furthermore, complete IFN-γ deficiency is characterized by a selective predisposition to infections caused by mycobacteria, *Salmonella*, or *Candida* species [123, 124]. This does not correlate with the clinical phenotype of MDS patients who typically show purulent bronchitis caused by bacteria which are capable of building a capsule such as *Streptococcus pneumoniae* or *Haemophilus influenzae* [76]. In the so far published cases of MDS, an infection with mycobacteria was only described once [76].

Besides its role for canonical NF-κB signaling, IRAK1 controls the induction of interferons via interferon regulatory factor 7 (IRF7) [109, 111, 114]. In human IRF7 deficiency, individuals are selectively susceptible to severe infections by influenza and SARS-CoV-2 and show an impaired type I IFN signature [125, 126]. In vitro, IRAK-1 regulates the transcriptional activation of IRF7 by directly binding and phosphorylating it. TLR7- and TLR9-mediated IFNα production is abolished in IRAK1-deficient mice, whereas inflammatory cytokine production is not impaired [111]. This brings up the question whether duplication of the *IRAK1* gene and thus IRAK1 overexpression causes an increased activation of the TLR7- and TLR9-mediated interferon-α induction pathway leading to an increased release of interferons and consequently to a hyperinflammatory immune response. However, CD169 expression on monocytes, which is correlated with systemic type I IFN levels, was normal in P3 both while he suffered from an infection and when he was free of infections [127, 128]. Further, MECP2-overexpressing mice had been described as particularly susceptible for severe influenza A infection. During infection, they show neutrophilia, increased cytokine production, excessive corticosterone levels, defective adaptive immunity, and vascular pathology. This raises the question if the inflammation-underlying pathomechanism in humans suffering from *MECP2* duplication syndrome is rather caused by the overexpression of MECP2 than the overexpression of IRAK1 [107]. In a humanized mouse model of MDS, intracerebroventricular antisense oligonucleotide (ASO) therapy was shown to decrease MECP2 expression in the brain and to reduce behavioral deficits as well as to restore/correct reduced IFN-γ mRNA levels in the blood [129]. If inflammation in MDS is rather caused by the duplication of *MECP2* itself, than by duplication of *IRAK1*, ASO against *MECP2* might be a feasible treatment option for these patients. The effects of such ASO therapy, applied in compartments such as the blood and lungs, may also warrant further investigation.

In summary, patients with *MECP2* duplication syndrome do not show increased canonical NF-κB signaling in whole blood, PBMCs, or SV40-immortalized fibroblasts. Therefore, we assume that these patients do not benefit from a therapeutic suppression of this pathway.

## Supplementary Information

Below is the link to the electronic supplementary material.

Supplementary file1 (DOCX 1016 KB)

## Data Availability

The datasets generated during the current study are available from the corresponding author on reasonable request.
