# Supplementary material for: IRAK1 Duplication in MECP2 Duplication Syndrome Does Not Increase Canonical NF-κB–Induced Inflammation
Source: J Clin Immunol. 2022 Nov 2;43(2):421–39. doi: 10.1007/s10875-022-01390-7 (PMC9628328; doi:10.1007/s10875-022-01390-7)

Electronic supplemental material for

***IRAK1* Duplication in *MECP2* Duplication Syndrome Does Not Increase Canonical NF-κB-Induced Inflammation**

Ilona Gottschalk^1,2^, Uwe Kölsch^3^, Dimitrios L. Wagner^2,4,5,6^, Jonas Kath^2,4^, Stefania Martini^2^, Renate Krüger^1^, Anne Puel^7,8^, Jean-Laurent Casanova^7,8,9,10,11^, Aleksandra Jezela-Stanek^12^, Rainer Rossi^13^, Salima El Chehadeh^14^, Hilde Van Esch^15^, Horst von Bernuth^1,2,3,16^

^1^ Department of Pediatric Respiratory Medicine, Immunology and Critical Care Medicine, Charité - Universitätsmedizin Berlin, corporate member of Freie Universität Berlin, Humboldt-Universität zu Berlin, and Berlin Institute of Health (BIH), Berlin, Germany

^2^ BIH Center for Regenerative Therapies (BCRT), Berlin Institute of Health (BIH), Berlin, Germany

^3^ Labor Berlin GmbH, Department of Immunology, Berlin, Germany

^4^ Berlin Center for Advanced Therapies (BeCAT), Charité - Universitätsmedizin Berlin, corporate member of Freie Universität, Berlin, Humboldt-Universität zu Berlin, and Berlin Institute of Health (BIH), Berlin, Germany

^5^ Institute of Transfusion Medicine, Charité - Universitätsmedizin Berlin, corporate member of Freie Universität Berlin, Humboldt-Universität zu Berlin, and Berlin Institute of Health (BIH), Berlin, Germany

^6^ Institute of Medical Immunology, Campus Virchow-Klinikum, Charité - Universitätsmedizin Berlin, corporate member of Freie Universität Berlin, Humboldt-Universität zu Berlin, and Berlin Institute of Health (BIH), Berlin, Germany

^7^ Laboratory of Human Genetics of Infectious Diseases, Necker Branch, INSERM U1163, Necker Hospital for Sick Children, Paris, France

^8^ University of Paris, Imagine Institute, Paris, France

^9^ St. Giles Laboratory of Human Genetics of Infectious Diseases, Rockefeller Branch, The Rockefeller University, New York, NY, USA

^10^ Howard Hughes Medical Institute, New York, NY, USA

^11^ Pediatric Hematology and Immunology Unit, Necker Hospital for Sick Children, AP-HP, Paris, France

^12^ Department of Genetics and Clinical Immunology, National Institute of Tuberculosis and Lung Diseases, Warsaw, Poland

^13^ Childrens’ Hospital Neukölln, Vivantes GmbH, Berlin, Germany

^14^ Institute of Medical Genetics of Alsace (IGMA), Strasbourg, France

^15^ Center for Human Genetics, University Hospitals Leuven, Leuven, Belgium

^16^ Berlin Institute of Health at Charité – Universitätsmedizin Berlin, Berlin, Germany

*Corresponding author:*

Horst von Bernuth, MD PhD

Department of Pediatric Respiratory Medicine, Immunology and Critical Care Medicine

Labor Berlin GmbH, Department of Immunology

Berlin Institute of Health (BIH), Berlin-Brandenburg Center for Regenerative Therapies (BCRT)

Charité – Universitätsmedizin Berlin

Augustenburger Platz 1

D-13353 Berlin

E-Mail: [horst.von-bernuth@charite.de](mailto:horst.von-bernuth@charite.de)

This file includes:

- Case Reports (P1-2, P4-9)
- Supplemental Tables S1, S2, S3
- Supplemental Figures S1, S2

**Case Reports**

Patient 1 was previously published as P14 in Bauer et al. [1]. The boy was diagnosed at the age of 11 and showed a de-novo-duplication in Array-CGH of 500 kb at Xq28. He suffered from infantile hypotonia and spoke only 4 words. He was able to walk independently at the age of 3.5 but lost this ability at the age of 10 so that he then needed a wheelchair. Since the age of 10, he suffered from a therapy resistant epilepsy. Furthermore, his mother reported feeding difficulties which deteriorated due to the epilepsy, but no gastroesophageal reflux. Throughout his life he had 18 severe infections which required intravenous antibiotic treatment including 14 episodes of bronchopneumonia. During his infections he showed fever up to 39.5°C. He deceased at the age 16 due to respiratory insufficiency in the context of total atelectasis and infection with pseudomonas. From the age of 12 on he received antibiotic prophylaxis. He was followed up twice a year by a neuropediatric specialist.

Patient 2 was described previously [P15 in 1, P1 in 2]. He was born after an uneventful pregnancy as the first child to healthy non-consanguineous parents. Family history was negative. At birth, he was very hypotonic with feeding problems. His development was severely delayed, with sitting at the age of 2 years and walking with support at the age of 3 years and half. He did not develop any active speech and shows repetitive behavior and hand flapping when he is excited. He suffered from recurrent infections occurring since the first months of life, necessitating almost continuous antibiotic therapy and frequent hospitalizations. At the age of 6 years, he was ventilated for 1 week because of serious pneumonia. After this period, he lost ambulation and his epilepsy became refractory. He was hospitalized multiple times because of pneumonia and respiratory distress with worsening of his general status. He developed a severe spasticity with contractures in upper and lower limbs. MRI of the brain showed global cerebral atrophy and atrophic hippocampi. He died at the age of 15 years because of respiratory failure. Array-CGH at the age of 2 years showed a very small duplication of 380 kb at Xq28. His mother is a carrier of the duplication.

Patient 4 was previously described as P13 in Bauer et al. and carries a very large duplication of 320 kb at Xq28 [1]. Unfortunately, we could not obtain current information about the boy.

Patient 5 is a 21-year-old male who was recently diagnosed with *MECP2* duplication syndrome and was not published before. He is a sibling of a non-identical twin pregnancy and showed infantile hypotonia and feeding problems. His development was severely delayed with absence of speech and walking at the age of 6 years. He is still ambulatory with aid. He developed absence epilepsy at the age of 14. Since birth he suffered from recurrent and severe infections (more than 32 episodes of pneumonia, 1 sepsis and at least 1 urinary tract infection) which regularly required hospitalization for intravenous antibiotic treatment. *S. aureus*, *H. influenzae B* and *Moxarella catarrhalis* were detected as causing pathogens in a bronchoalveolar lavage. Infections are usually associated with high fever. Additionally, he has 4 to 5 unexplainable episodes of fever per year. Immunoglobulins A and M are within the normal range, whereas immunoglobulin G is above the range showing an isolated elevation of IgG3. The boy receives azithromycin as an antibiotic prophylaxis and is followed up by a multidisciplinary team once a year. He carries a complex chromosomal aberration including a terminal deletion of the PAR1 region at Xp22.33 as well as a duplication of 3.3 Mb at Xq28. His mother is an asymptomatic carrier and had a brother who died at young age with a highly similar phenotype.

Patient 6 is a 10-year-old boy who was not described before. He is the third child of healthy parents born after an uneventful pregnancy and a negative family history. At birth, intermittent myoclonic seizures were noted as well as important feeding problems. His development was severely delayed with severe hypotonia. He never learned to walk and did not acquire any speech. Between the age of 2 and 5 he suffered from 5 epsiodes of severe infections requiring intravenous antibiotic treatment, of which at least 3 were bronchopneumonia. So far, his recurrent infections are treatable in an ambulatory setting. He has no history of sepsis, meningitis or purulent otitis. During his episodes of infection he showed high fever up to 40.5°C. His parents also report 4 to 5 unexplainable epsiodes of fever anually. IgG, A and M were within normal range. Analysis of IgG subclasses showed elevated IgG2. A bronchoalveolar lavage was never performed. At the age of 9 years, he started to have tonic seizures, refractory to multi-anti-epileptic therapy. MRI of the brain showed hypoplasia of the corpus callosum and atrophy of the frontal lobe. The boy is assesed by a geneticist once a year as well as on an irregular basis by a neurologist and pneumologist. He does not receive antibiotic prophylaxis or IgG substitution. Array-CGH showed a very large de novo terminal duplication of 7 Mb at Xq28.

Patient 7 is a 5-year-old boy who was not published before. Array-CGH showed a large de-novo duplication of 3 Mb at Xq28. He suffers from muscular hypotonia and needs support to walk. His speech is limited to a few words. Shortly after birth, feeding problems were noted. The diagnosis of gastro-esophageal reflux leaded to medication starting at the age of 3 years. No epilepsy was reported but he showed an abnormal EEG. Furthermore, pituitary hormone deficiencies, an atrial septum defect, craniosynostosis and cerebral cysts were diagnosed. In his first 3 years of life, he suffered from 5 to 6 lower airway infections including at least 4 episodes of pneumonia and 2 urinary tract infections at the age of 3 months and 15 months. In total, he was diagnosed with pneumonia 6 times, the first one developed at the age of 6 months. One of the infections was a methicillin-susceptible *Staphylococcus aureus* (MSSA)-pneumonia with sepsis. A bronchoalveolar lavage was never performed but *S. aureus* and alpha-hemolytic streptococci were detected in blood. His parents reported unexplainable episodes of fever (~40°C) up to five times per year. IgG, A and M and IgG subclasses were within normal range. The boy is followed up by a multidisciplinary team (General Pediatrics, Neurology, Pulmonology, Endocrinology) twice a year. He does not receive any prophylactic treatment.

Patient 8 is an unpublished 10-year-old boy from Germany. Array-CGH showed a combination of duplication and triplication of 590 kb at Xq28. His mother is an asymptomatic carrier. At birth, muscular hypotonia was noted. He has impaired speech (10-15 words) but good social interaction. At the age of 1, Obstructive Sleep Apnea Syndrome (OSAS) was diagnosed and required breathing therapy and intubation leading to a difficult weaning process. He was able to walk at the age of 2. So far, no epilepsy was reported. A X-ray-proven bronchopneumonia at the age of 11 months required hospitalization but no antibiotic treatment. Since then, recurrent infections of the upper and less frequently lower respiratory tract are apparent especially in winter months, rarely requiring antibiotic treatment. In one episode of bronchitis, *respiratory syncytial virus* was detected. He shows good antibody titers against pneumococci and IgG, A and M are within normal range. The boy is followed up by a multidisciplinary team (sociopediatric center, ENT, orthopedics, ophthalmology) twice a year. He does not receive any prophylactic treatment as well.

Patient 9 is a 10-year-old boy from Germany with a de-novo duplication of 1.3 Mb at Xq28 who was previously published [P28 in 1]. He is severely developmentally delayed. Starting at the age of 9, he developed recurrent infections summing up to 7 episodes of pneumonia, 1 purulent otitis and 5 urinary tract infections. 3 of the 7 episodes of pneumonia were X-ray-proven bronchopneumonia which required antibiotic treatment and 1 was an influenza B pneumonia. During these episodes of infections high CRP values over 200 mg/l were detected repeatedly, the maximum measured being 321 mg/l. On the other hand, the boy showed unexplainable episodes of fever up to max 41°C without CRP elevation 2-3 times per year. Several booster vaccinations were administered because of a lack of antibody production against pneumococci. A regular follow up is not performed.

**References**

[1] Bauer M, Kölsch U, Krüger R, Unterwalder N, Hameister K, Kaiser FM, et al. Infectious and immunologic phenotype of MECP2 duplication syndrome. J Clin Immunol 2015;35:168–81. https://doi.org/10.1007/s10875-015-0129-5.

[2] Nageshappa S, Carromeu C, Trujillo CA, Mesci P, Espuny-Camacho I, Pasciuto E, et al. Altered neuronal network and rescue in a human MECP2 duplication model. Mol Psychiatry 2016;21:178–88. https://doi.org/10.1038/mp.2015.128.

**Table S1** Isolated Pathogens in published patients with *MECP2* duplication syndrome [1–7]

| Pathogen | Specification |
| --- | --- |
| 45 bacteria | - 8 *S.pneumoniae* - 5 *H.influenzae* - 5 *E.coli* - 4 *S.aureus* - 3 *P. aeruginosa* - 2 *P.mirabilis* - 2 *Streptococcus group A* - *Stenotrophomonas maltophilia* - *Enterobacter tracheitis* - *S.agalactiae* - *Streptococcus group C* - *Streptococcus group F* - *S.epidermidis* - *K.pneumonia* - *K.oxytoca* - *M. tuberculosis* - *O.anthropi* - *A.hydrophila* - *C.freundii* - *B.fragilis* - *S.enteritidis* - *S.marcescens* - *S.plymuthica* |
| 6 viruses | - 3 *Respiratory syncytial viruses* - *Influenza A* - *Influenza B* - *Rhinovirus* |
| 4 fungi | - 4 *Candida albicans* |

**References**

[1] Bialer M. De novo trisomy Xq28-qter detected by subtelomeric FISH screening. Am J Hum Genet 2003.

[2] Reardon W, Donoghue V, Murphy A-M, King MD, Mayne PD, Horn N, et al. Progressive cerebellar degenerative changes in the severe mental retardation syndrome caused by duplication of MECP2 and adjacent loci on Xq28. Eur J Pediatr 2010;169:941–9. https://doi.org/10.1007/s00431-010-1144-4.

[3] Shimada S, Okamoto N, Hirasawa K, Yoshii K, Tani Y, Sugawara M, et al. Clinical manifestations of Xq28 functional disomy involving MECP2 in one female and two male patients. Am J Med Genet A 2013;161A:1779–85. https://doi.org/10.1002/ajmg.a.35975.

[4] Carvalho CMB, Ramocki MB, Pehlivan D, Franco LM, Gonzaga-Jauregui C, Fang P, et al. Inverted genomic segments and complex triplication rearrangements are mediated by inverted repeats in the human genome. Nat Genet 2011;43:1074–81. https://doi.org/10.1038/ng.944.

[5] Bauer M, Krüger R, Kölsch U, Unterwalder N, Meisel C, Wahn V, et al. Antibiotic Prophylaxis, Immunoglobulin Substitution and Supportive Measures Prevent Infections in MECP2 Duplication Syndrome. Pediatr Infect Dis J 2018;37:466–8. https://doi.org/10.1097/INF.0000000000001799.

[6] Bauer M, Kölsch U, Krüger R, Unterwalder N, Hameister K, Kaiser FM, et al. Infectious and immunologic phenotype of MECP2 duplication syndrome. J Clin Immunol 2015;35:168–81. https://doi.org/10.1007/s10875-015-0129-5.

[7] van Baelen A, Verhoustraeten L, Kenis S, Meuwissen M, Boudewyns A, van Hoorenbeeck K, et al. Sleep-disordered breathing and nocturnal hypoventilation in children with the MECP2 duplication syndrome: A case series and review of the literature. Am J Med Genet A 2020. https://doi.org/10.1002/ajmg.a.61790.

**Table S2** Quantitation of western blot from Fig. 3. IRAK1 and GAPDH protein signals were normalized to the C1 lane using Image Lab.

|  | IRAK1 signal | GAPDH signal |
| --- | --- | --- |
| C1 | 1 | 1 |
| C3 | 1,262209 | 0,73088 |
| C5 | 1,346706 | 0,698614 |
| C6 | 1,023595 | 1,225337 |
| P1 | 3,200311 | 0,764149 |
| P2 | 2,715603 | 0,732794 |
| P3 | 4,355269 | 1,181709 |
| P4 | 3,606163 | 0,767468 |
| *IRAK1 y/-* |  | 1,047704 |
| *IRAK4 -/-* | 1,414804 | 1,064115 |

**Table S3** Exact boundaries of duplications and triplications of Xq28 in the 9 patients (GRCh37/hg19) and genes included in the respective regions according to genome.ucsc.edu. Triplicated regions are marked in bold.

| Patient | P1 | P2 | P3 | P4 |
| --- | --- | --- | --- | --- |
| Duplicated/ **triplicated** region | chrX:152,435,900 -153,564,230 | chrX:153,155,029 -153,529,964 | chrX:153,046,806 -153,364,010 | chrX:152,916,694 -153,414,333 |
| Genes included in the region | *MAGEA1*  *ZNF275*  *ZFP92*  *TREX2*  *HAUS7*  *BGN*  *ATP2B3*  *CCNQ*  *DUSP9*  *PNCK*  *SLC6A8*  *BCAP31*  *ABCD1*  *PLXNB3*  *SRPK3*  *IDH3G*  *SSR4*  *PDZD4*  *L1CAM*  *AVPR2*  *ARHGAP4*  *NAA10*  *RENBP*  *DL490658*  *HCFC1*  *TMEM187*  *MIR3202-1*  *MIR3202-2*  *IRAK1*  *MIR718*  *MECP2*  *OPN1LW*  *TEX28*  *OPN1MW*  *TEX28*  *OPN1MW3*  *TEX28*  *TKTL1* | *AVPR2*  *ARHGAP4*  *NAA10*  *RENBP*  *DL490658*  *HCFC1*  *TMEM187*  *MIR3202-1*  *MIR3202-2*  *IRAK1*  *MIR718*  *MECP2*  *OPN1LW*  *TEX28*  *OPN1MW*  *TEX28*  *OPN1MW3*  *TEX28*  *TKTL1* | *SRPK3*  *IDH3G*  *SSR4*  *PDZD4*  *L1CAM*  *AVPR2*  *ARHGAP4*  *NAA10*  *RENBP*  *DL490658*  *HCFC1*  *TMEM187*  *MIR3202-1*  *MIR3202-2*  *IRAK1*  *MIR718*  *MECP2* | *DUSP9*  *PNCK*  *SLC6A8*  *BCAP31*  *ABCD1*  *PLXNB3*  *SRPK3*  *IDH3G*  *SSR4*  *PDZD4*  *L1CAM*  *AVPR2*  *ARHGAP4*  *NAA10*  *RENBP*  *DL490658*  *HCFC1*  *TMEM187*  *MIR3202-1*  *MIR3202-2*  *IRAK1*  *MIR718*  *MECP2*  *OPN1LW* |

| Patient | P5 | P6 | P7 | P8 | P9 |
| --- | --- | --- | --- | --- | --- |
| Duplicated/ **triplicated** region | chrX:151,963,529 -155,233,098  **triplicated:**  **chrX: 154,964,042-155,233,098** | chrX:147,824,471 -154,929,305 | chrX:152,129,407 -155,232,894 | chrX:152,937,102 -153,523,169  **triplicated:**  **chrX: 152,949,788-153,505,538** | chrX:152,192,816 -153,531,883 |
| Genes included in the region | *CETN2*  *NSDHL*  *ZNF185*  *PNMA5*  *PNMA3*  *PNMA6A*  *PNMA6D*  *PNMA6A*  *MAGEA1*  *ZNF275*  *ZFP92*  *TREX2*  *HAUS7*  *BGN*  *ATP2B3*  *CCNQ*  *DUSP9*  *PNCK*  *SLC6A8*  *BCAP31*  *ABCD1*  *PLXNB3*  *SRPK3*  *IDH3G*  *SSR4*  *PDZD4*  *L1CAM*  *AVPR2*  *ARHGAP4*  *NAA10*  *RENBP*  *DL490658*  *HCFC1*  *TMEM187*  *MIR3202-1*  *MIR3202-2*  *IRAK1*  *MIR718*  *MECP2*  *OPN1LW*  *TEX28*  *OPN1MW*  *TEX28*  *OPN1MW3*  *TEX28*  *TKTL1*  *FLNA*  *EMD*  *XX-FW83563B9.5*  *RPL10*  *SNORA70*  *DNASE1L1*  *TAZ*  *CH17-340M24.3*  *ATP6AP1*  *GDI1*  *FAM50A*  *PLXNA3*  *LAGE3*  *UBL4A*  *SLC10A3*  *FAM3A*  *G6PD*  *IKBKG*  *FAM223A*  *CTAG1A*  *CTAG1B*  *FAM223B*  *IKBKG*  *CTAG2*  *GAB3*  *DKC1*  *SNORA36A*  *SNORA56*  *MPP1*  *SMIM9*  *F8*  *H2AB1*  *F8A1*  *MIR1184-1*  *FUNDC2*  *CMC4*  *MTCP1*  *BRCC3*  *VBP1*  *RAB39B*  *CLIC2*  *RP13-228J13.1*  *H2AB2*  *F8A2*  *MIR1184-2*  *F8A3*  *MIR1184-3*  *H2AB3*  *TMLHE-AS1*  *RP11-218L14.4*  *TMLHE*  ***SPRY3***  ***VAMP7***  ***IL9R*** | *AFF2*  *IDS*  *AF011889.2*  *AF011889.5*  *EOLA1*  *MAGEA9B*  *HSFX2*  *TMEM185A*  *MAGEA11*  *TMEM185A*  *TMEM185A*  *HSFX1*  *MAGEA9*  *MAGEA8-AS1*  *MAGEA8*  *EOLA2*  *LINC00894*  *MIR2114*  *MAMLD1*  *MTM1*  *MTMR1*  *CD99L2*  *HMGB3*  *MIR4330*  *GPR50*  *VMA21*  *PASD1*  *PRRG3*  *FATE1*  *CNGA2*  *MAGEA4*  *GABRE*  *MIR452*  *MAGEA10-MAGEA5*  *MAGEA10*  *GABRA3*  *RNU6-764P*  *MIR105-1*  *Mir_105*  *Mir_105*  *MIR767*  *Mir_105*  *MIR105-2*  *GABRQ*  *MAGEA6*  *CSAG3*  *MAGEA2B*  *MAGEA12*  *CSAG1*  *CSAG1*  *MAGEA2*  *CSAG2*  *MAGEA3*  *CETN2*  *NSDHL*  *ZNF185*  *PNMA5*  *PNMA3*  *PNMA6A*  *PNMA6D*  *PNMA6A*  *MAGEA1*  *ZNF275*  *ZFP92*  *TREX2*  *HAUS7*  *BGN*  *ATP2B3*  *CCNQ*  *DUSP9*  *PNCK*  *SLC6A8*  *BCAP31*  *ABCD1*  *PLXNB3*  *SRPK3*  *IDH3G*  *SSR4*  *PDZD4*  *L1CAM*  *AVPR2*  *ARHGAP4*  *NAA10*  *RENBP*  *DL490658*  *HCFC1*  *TMEM187*  *MIR3202-1*  *MIR3202-2*  *IRAK1*  *MIR718*  *MECP2*  *OPN1LW*  *TEX28*  *OPN1MW*  *TEX28*  *OPN1MW3*  *TEX28*  *TKTL1*  *FLNA*  *EMD*  *XX-FW83563B9.5*  *RPL10*  *SNORA70*  *DNASE1L1*  *TAZ*  *CH17-340M24.3*  *ATP6AP1*  *GDI1*  *FAM50A*  *PLXNA3*  *LAGE3*  *UBL4A*  *SLC10A3*  *FAM3A*  *G6PD*  *IKBKG*  *FAM223A*  *CTAG1A*  *CTAG1B*  *FAM223B*  *IKBKG*  *CTAG2*  *GAB3*  *DKC1*  *SNORA36A*  *SNORA56*  *MPP1*  *SMIM9*  *F8*  *H2AB1*  *F8A1*  *MIR1184-1*  *FUNDC2*  *CMC4*  *MTCP1*  *BRCC3*  *VBP1*  *RAB39B*  *CLIC2*  *RP13-228J13.1*  *H2AB2*  *F8A2*  *MIR1184-2*  *F8A3*  *MIR1184-3*  *H2AB3*  *TMLHE-AS1*  *RP11-218L14.4*  *TMLHE* | *ZNF185*  *PNMA5*  *PNMA3*  *PNMA6A*  *PNMA6D*  *PNMA6A*  *MAGEA1*  *ZNF275*  *ZFP92*  *TREX2*  *HAUS7*  *BGN*  *ATP2B3*  *CCNQ*  *DUSP9*  *PNCK*  *SLC6A8*  *BCAP31*  *ABCD1*  *PLXNB3*  *SRPK3*  *IDH3G*  *SSR4*  *PDZD4*  *L1CAM*  *AVPR2*  *ARHGAP4*  *NAA10*  *RENBP*  *DL490658*  *HCFC1*  *TMEM187*  *MIR3202-1*  *MIR3202-2*  *IRAK1*  *MIR718*  *MECP2*  *OPN1LW*  *TEX28*  *OPN1MW*  *TEX28*  *OPN1MW3*  *TEX28*  *TKTL1*  *FLNA*  *EMD*  *XX-FW83563B9.5*  *RPL10*  *SNORA70*  *DNASE1L1*  *TAZ*  *CH17-340M24.3*  *ATP6AP1*  *GDI1*  *FAM50A*  *PLXNA3*  *LAGE3*  *UBL4A*  *SLC10A3*  *FAM3A*  *G6PD*  *IKBKG*  *FAM223A*  *CTAG1A*  *CTAG1B*  *FAM223B*  *IKBKG*  *CTAG2*  *GAB3*  *DKC1*  *SNORA36A*  *SNORA56*  *MPP1*  *SMIM9*  *F8*  *H2AB1*  *F8A1*  *MIR1184-1*  *FUNDC2*  *CMC4*  *MTCP1*  *BRCC3*  *VBP1*  *RAB39B*  *CLIC2*  *RP13-228J13.1*  *H2AB2*  *F8A2*  *MIR1184-2*  *F8A3*  *MIR1184-3*  *H2AB3*  *TMLHE-AS1*  *RP11-218L14.4*  *TMLHE*  *SPRY3*  *VAMP7*  *IL9R* | *PNCK*  ***SLC6A8***  ***BCAP31***  ***ABCD1***  ***PLXNB3***  ***SRPK3***  ***IDH3G***  ***SSR4***  ***PDZD4***  ***L1CAM***  ***AVPR2***  ***ARHGAP4***  ***NAA10***  ***RENBP***  ***DL490658***  ***HCFC1***  ***TMEM187***  ***MIR3202-1***  ***MIR3202-2***  ***IRAK1***  ***MIR718***  ***MECP2***  ***OPN1LW***  ***TEX28***  ***OPN1MW***  ***TEX28***  ***OPN1MW3***  ***TEX28*** | *PNMA3*  *PNMA6A*  *PNMA6D*  *PNMA6A*  *MAGEA1*  *ZNF275*  *ZFP92*  *TREX2*  *HAUS7*  *BGN*  *ATP2B3*  *CCNQ*  *DUSP9*  *PNCK*  *SLC6A8*  *BCAP31*  *ABCD1*  *PLXNB3*  *SRPK3*  *IDH3G*  *SSR4*  *PDZD4*  *L1CAM*  *AVPR2*  *ARHGAP4*  *NAA10*  *RENBP*  *DL490658*  *HCFC1*  *TMEM187*  *MIR3202-1*  *MIR3202-2*  *IRAK1*  *MIR718*  *MECP2*  *OPN1LW*  *TEX28*  *OPN1MW*  *TEX28*  *OPN1MW3*  *TEX28*  *TKTL1* |

**Figure S1** Episodes of pneumonia in P3 per year of life. The time when we started crucial therapeutic strategies are indicated.


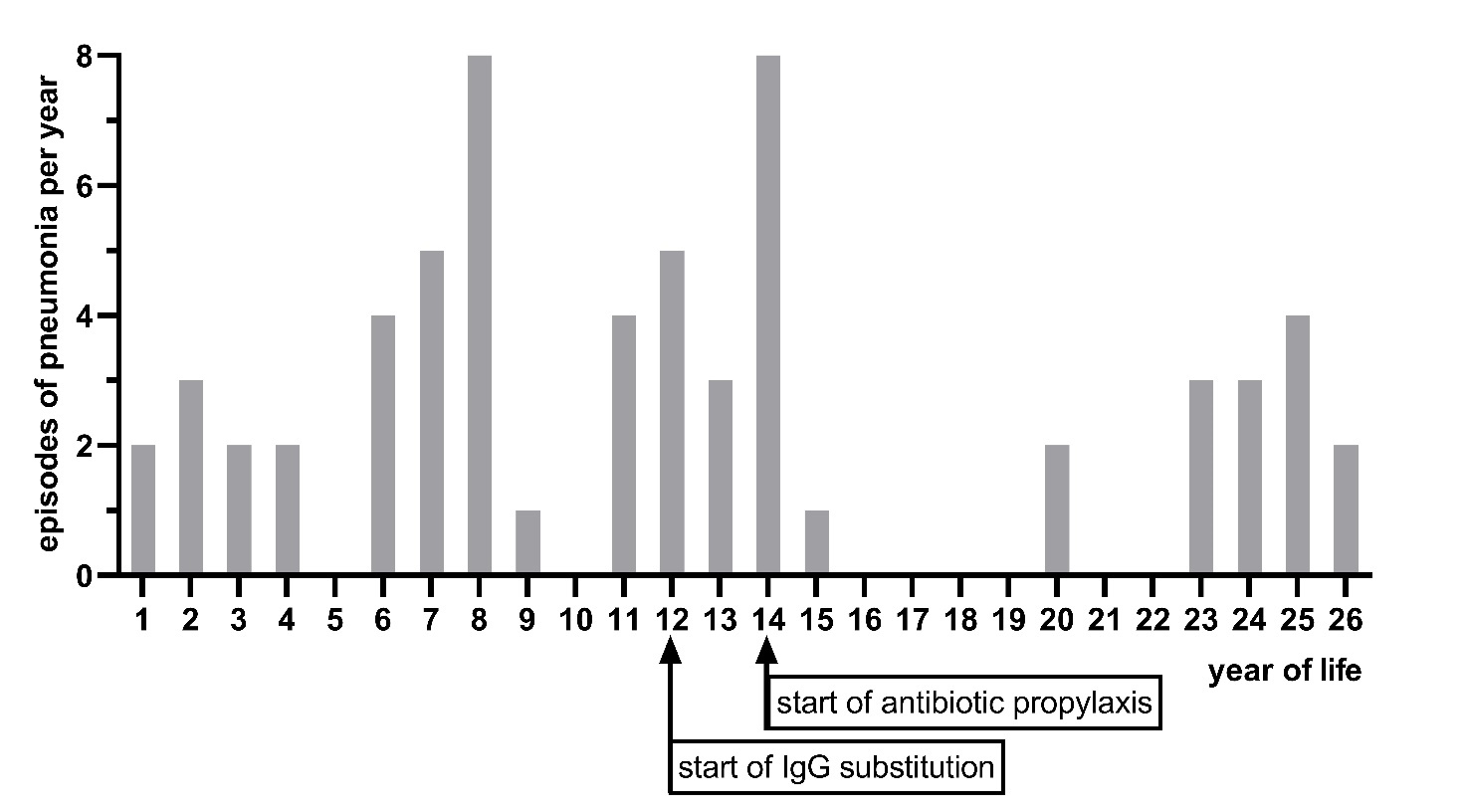


**Figure S2** Latest CT scan of P3 at the age of 25 showing ubiquitous bronchiectasis, leading in the lower lobes on both sides, with partially bullous destructive pulmonary changes in the left lower lobe and incipient in the right upper lobe. Central secretions of the bronchial system and subsegmental mucus plugging. Associated (incipient) infiltrates. Peribronchiovascular as well as subpleural opacities and reticular patterns fitting to condition after aspiration.


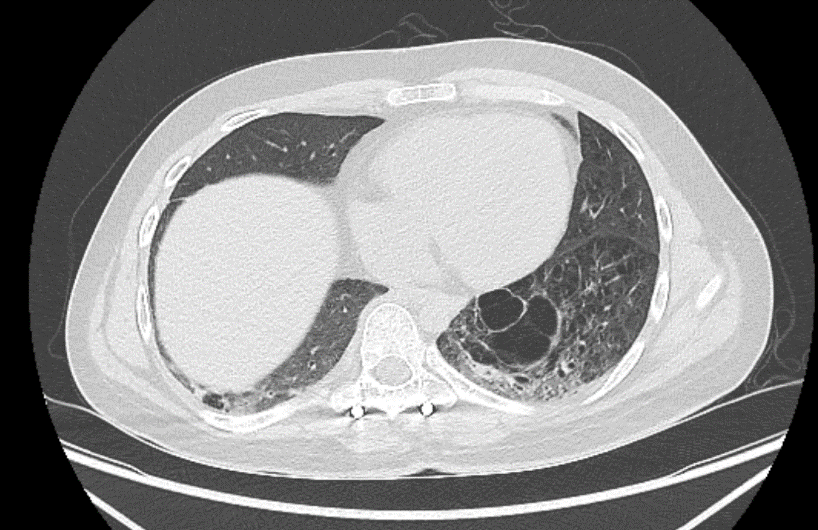

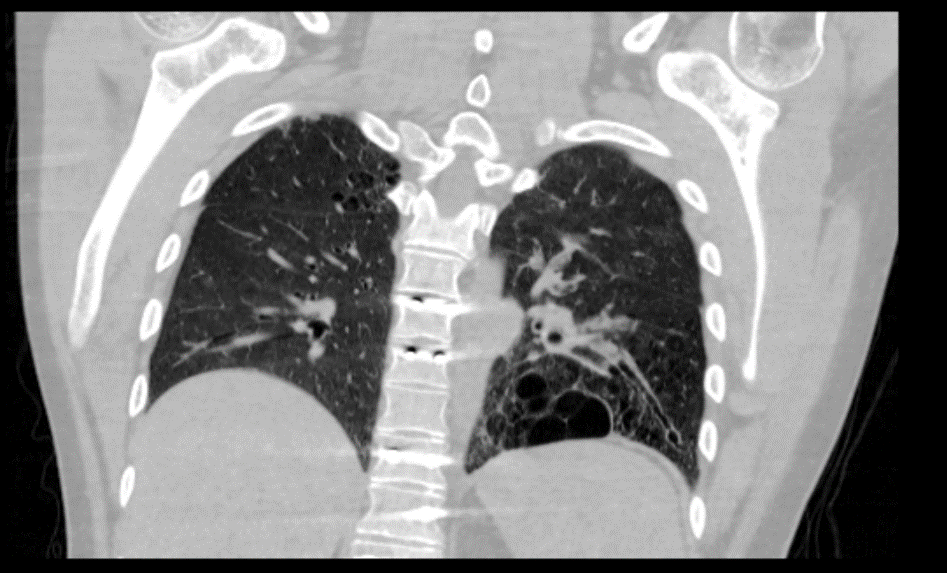


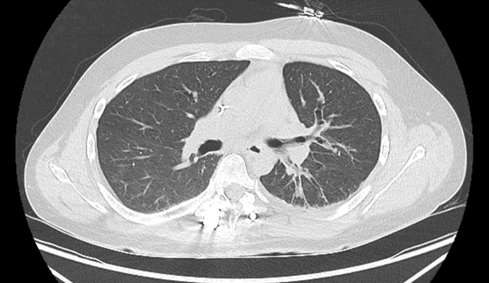

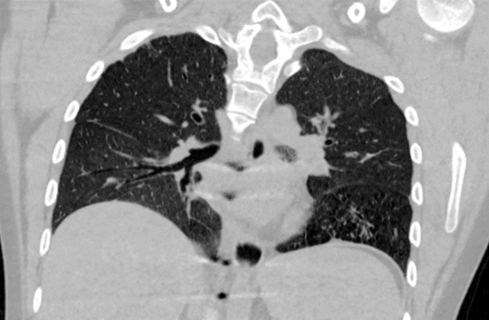

Supplement: Supplementary file 1 — Supplementary file1 (DOCX 1016 KB) [file 10875_2022_1390_MOESM1_ESM.docx]
